# Exploring how material cues drive sensorimotor prediction across different levels of autistic-like traits

**DOI:** 10.1007/s00221-019-05586-z

**Published:** 2019-06-27

**Authors:** Tom Arthur, Sam Vine, Mark Brosnan, Gavin Buckingham

**Affiliations:** 1 Sport and Health Sciences, College of Life and Environmental Sciences, University of Exeter, St Luke’s Campus, Heavitree Road, Exeter, EX1 2LU Devon UK; 2 Department of Psychology, University of Bath, Bath, BA2 7AY UK

**Keywords:** Autism, Movement, Object lifting, Weight illusion, Grip force

## Abstract

Recent research proposes that sensorimotor difficulties, such as those experienced by many autistic people, may arise from atypicalities in prediction. Accordingly, we examined the relationship between non-clinical autistic-like traits and sensorimotor prediction in the material-weight illusion, where prior expectations derived from material cues typically bias one’s perception and action. Specifically, prediction-related tendencies in perception of weight, gaze patterns, and lifting actions were probed using a combination of self-report, eye-tracking, motion-capture, and force-based measures. No prediction-related associations between autistic-like traits and sensorimotor control emerged for any of these variables. Follow-up analyses, however, revealed that greater autistic-like traits were correlated with reduced adaptation of gaze with changes in environmental uncertainty. These findings challenge proposals of gross predictive atypicalities in autistic people, but suggest that the dynamic integration of prior information and environmental statistics may be related to autistic-like traits. Further research into this relationship is warranted in autistic populations, to assist the development of future movement-based coaching methods.

**Electronic supplementary material:**

The online version of this article (10.1007/s00221-019-05586-z) contains supplementary material, which is available to authorized users.

## Introduction

Sensorimotor atypicalities are increasingly being viewed as ‘cardinal’ feature of Autism Spectrum Disorder (ASD), which impact on lifelong living proficiencies, social development, and quality of life (Fournier et al. 2010; Gowen and Hamilton 2013). Indeed, movement-related difficulties are experienced by most autistic people (for review, see Gowen and Hamilton 2013), with postural abnormalities, sensory hypersensitivities, and impairments in skills requiring gross and/or fine motor co-ordination all commonplace (Fournier et al. 2010). While these features rarely necessitate medical treatment, they contribute to substantial practical, financial, and health-related hardships (Buescher et al. 2014; Pellicano et al. 2014). For example, movement-based difficulties in autism may underpin reduced motivation and participation in physical activity (Leary and Hill 1996; Scharoun et al. 2017). These difficulties also can precede, and even predict various aptitudes in childhood and adult life (e.g., daily living skills, social skills, Jasmin et al. 2009; Brandwein et al. 2015). Consequently, research into the aetiology and management of these abilities is demanded both by academics (Gowen and Hamilton 2013) and the autism community (Pellicano et al. 2014).

Emerging research suggests that these sensorimotor difficulties stem from atypical predictive processing, with autistic people proposed to utilise prior information less accurately and/or efficiently (e.g., Pellicano and Burr 2012; Gomot and Wicker 2012; Friston et al. 2013; Sinha et al. 2014; Van de Cruys et al. 2014). Sensorimotor control involve complex, co-ordinated contributions from various distinct subcomponents (e.g., cognitive, visual, and motor systems), which respond to ‘bottom–up’ (stimulus-driven) informational sources and internally driven (‘top–down’) predictive models (Corbetta et al. 2008; Land 2009). Abnormalities in ‘top–down’ control can limit the performance and learning of goal-directed actions (Kording et al. 2007; Land 2009) and may exemplify a ‘shared endophenotype’ that underpins socio-behavioral difficulties in autism (e.g., social-communication deficits, repetitive behaviors, and attention to detail; Pellicano and Burr 2012; Sinha et al. 2014). Indeed, in motor control studies, autistic individuals show impaired postural adjustments in anticipation of changes in object load (Schmitz et al. 2003) and inaccurate initial force outputs during precision-grip actions (Mosconi et al. 2013; Wang et al. 2015), effects which signal an increased reliance on ‘bottom–up’ (as opposed to ‘top–down’) sensory information. Similarly, prediction-related differences emerge in cognition and visual processing, with autistic individuals demonstrating diminished ‘top–down’ gaze adaptation in double-step saccade paradigms (Johnson et al. 2013; Mosconi et al. 2013) and abnormalities in prediction-related neural regions (e.g., the cerebellum, Frith 2003). Such ‘top–down’ limitations lead to greater employment of ‘bottom–up’ attentional (e.g., proprioception, visual feedback; Haswell et al. 2009) and neurobiological systems (e.g., Soulières et al. 2009), while co-vary with movement-related difficulties in autism (Mosconi et al. 2013).

However, feedforward atypicalities have not been consistently detected in research (Palmer et al. 2017; Tewolde et al. 2018). For example, autistic children exhibit typical rates of motor adaptation in various tasks that require, and depend on, broad abilities to utilise ‘top–down’ internal models (Gidley-Larson et al. 2008), while prediction-related atypicalities in perception (e.g., global processing; Brosnan et al. 2004) do not inevitably transfer onto action or behavior (Palmer et al. 2017). Similarly, the nature and severity of movement-related difficulties varies between individuals and empirical contexts (Green et al. 2002; Palmer et al. 2017). This has prompted suggestions that autism-related difficulties originate from finer, context-sensitive differences in the integration of predictive and environmental statistics (Lawson et al. 2014; Palmer et al. 2017), as opposed to generic attenuations in the use of prior expectations. Consequently, research must decipher which specific mechanisms are implicated in autism (Haker et al. 2016). To do this, illusion-based paradigms offer notable value, as they can highlight ‘top–down’ influences on the processing of ambiguous sensory information (Geisler and Kersten 2002; Brown and Friston 2012). Interestingly, although autistic people do appear less susceptible to some perceptual illusions (e.g., Happé 1996; Mitchell et al. 2010; Ropar and Mitchell 2002), results are mixed and often complicated by heterogeneity in sampling characteristics (Van der Hallen et al. 2015).

To address these empirical inconsistencies and better separate autism-specific atypicalities from potential confounds (e.g., cognitive ability, symptom severity, development, and comorbidities), recent research has explored how sensorimotor outcomes relate to autistic-like traits in general populations (Landry and Chouinard 2016). Autistic-like traits are behavioral characteristics such as social imperviousness, directness in conversation, lack of imagination, affinity for solitude, and difficulty displaying emotions (Gernsbacher et al. 2017), which can be readily indexed using self-report measures such as the Autism Spectrum Quotient (AQ: Baron-Cohen et al. 2001). Such autistic-like traits vary continuously across the general population, with ASD proposed to reside at the extreme end of this continuum (Baron-Cohen et al. 2001, 2006; Ruzich et al. 2015). Consequently, empirical links between self-reported autistic-like traits and behavioral variables have enabled researchers to identify various cognitive, perceptual, and social differences associated with autism (e.g., Almeida et al. 2014; Poljac et al. 2013; Cooper et al. 2013; Jameel et al. 2014).

Interestingly, higher levels of autistic-like traits have been shown to relate to reduced illusory effects in some non-clinical studies (e.g., Chouinard et al. 2013, 2016). Recently, from a sensorimotor perspective, Buckingham et al. (2016) explored links between autistic-like traits and predictive sensorimotor control during object lifting, using a Size–Weight Illusion (SWI) paradigm. In the SWI, small objects are experienced as feeling heavier more than larger ones of an equal mass (Charpentier 1891), an effect underpinned by the prior expectation that larger items tend to be heavier than smaller items (Buckingham 2014). Interestingly, no relationship emerged between autistic-like traits and the magnitude of this illusion, challenging the assumptions of broad autism-related atypicalities in prediction (e.g., Pellicano and Burr 2012). However, participants with higher levels of autistic-like traits showed reduced ‘top–down’ bias of movement, as indexed by differences in peak grip and load force rates between larger (heavy-looking) and smaller (lighter-looking) objects. These findings suggest that, despite being equally susceptible to the perceptual SWI, high-trait individuals are less inclined to utilise prior information in their motor programmes, a dissociation which has also been reported for the rubber-hand illusion (Palmer et al. 2013, 2015).

The transferability of these results across movement-based contexts remains unclear, as observed relationships were weak (*R*^2^ = 0.06) and likely dependent on contextual factors. On one hand, ‘top–down’ expectations of weight influence lifting forces when objects differ in material, shape, and/or density (Gordon et al. 1991; Grandy and Westwood 2006; Buckingham et al. 2009). Similarly, abilities to regulate grip forces are influential in various daily living skills, including those known to be impaired in autism (e.g., dressing and writing; Fuentes et al. 2009; Wang et al. 2015). Conversely though, Buckingham et al. (2016)’s results may not necessarily reflect gross attenuations in the use of prior information, as lifting actions are directed by various cognitive (e.g., expected weight; Johansson and Westling 1988), attentional (e.g., vision; Gordon et al. 1991), and haptic (e.g., density; Grandy and Westwood 2006) mechanisms. Moreover, it is argued that something is unique about how volumetric features are processed in the brain (Saccone and Chouinard 2019), with the SWI underpinned by context-specific ‘top–down’ expectancies (i.e., predictions related to size-weight modelling; Buckingham and Goodale 2013) and haptic cues (e.g., object density; Buckingham 2014). As these processing tendencies are not entirely dependent on prior experience or knowledge (Saccone and Chouinard 2019), further scrutiny into the observed effects is warranted.

Therefore, we utilised a Material–Weight illusion (MWI) paradigm to better isolate associations between autistic-like traits and predictive sensorimotor control. Like the SWI, the MWI occurs when heavy-looking materials (e.g., granite) are perceived as feeling lighter, and lifted with greater initial force rates, than lighter-looking (e.g., polystyrene) items of the same mass (Wolfe 1898; Seashore 1899; Buckingham et al. 2009). Importantly, these effects are not driven by size-based expectations or low-level haptic cues (e.g., variations in centre of mass or density), but by prior expectations relating to material properties derived from prior experiences (Saccone and Chouinard 2019). Consequently, in line with predictive theories of autism (e.g., Pellicano and Burr 2012; Sinha et al. 2014) and previous illusory research (e.g., Chouinard et al. 2013), we hypothesised that the number of autistic-like traits that an individual presents will negatively correlate with the magnitude of the perceptual MWI.

Beyond our examination of fingertip forces, we conducted a multi-modal assessment of sensorimotor control to explore whether any abnormalities are broad and transferable across processing domains (Pellicano and Burr 2012), or whether they are underpinned by precise mechanisms (e.g., relating to environmental volatility, Lawson et al. 2017). Specifically, to extend Buckingham et al. (2016)’s previous findings, we probed expectation-related changes in both lifting forces and velocities between light- (polystyrene) and heavy-looking (granite) materials. Here, attenuations in ‘top–down’ control can be signalled via less-divergent lifting profiles (i.e., reduced expectation-based scaling of movement; Johansson and Westling 1988) and prolonged preparatory movements phases, which facilitate proprioceptive (i.e., ‘bottom–up’) interpretations of object mass (Hamilton et al. 2007). We also measured visual search rate and gaze fixations, as longer fixations prior to skill execution reflect extended periods of ‘top–down’ cognitive processing (Vickers 1996) and increases in search rate (i.e., shorter, more-frequent fixations) signal more stimulus-driven attentional control (Williams et al. 2002; Corbetta et al. 2008). On the basis of the aforementioned ‘domain-general’ theories (Pellicano and Burr 2012; Sinha et al. 2014) and Buckingham et al. (2016)’s data, which posit that socio-behavioral and movement-based difficulties in autism are both underpinned by atypical predictive processing, we estimated that ‘top–down’ sensorimotor control would be correlated with self-reported autistic-like traits. Specifically, greater autistic-like traits were hypothesised to co-vary with a reduced susceptibility to the perceptual MWI, attenuated expectation-based scaling of lifting force rate, prolonged preparatory movement kinematics, elevated visual search rates, and shorter gaze fixations prior to skill execution.

## Methods

### Participants

Ninety-two participants (47 males and 45 females; 23.10 ± 3.32 years) were recruited, the majority of whom (*n* = 83; 90%) were self-reported right handers. All were naïve to the study aims and had normal or corrected-to-normal vision. Participants reporting any condition known to affect sensorimotor control, including ASD, were excluded. One individual with developmental co-ordination disorder and one with prior injury were removed. The study received approval from the School of Sport and Health Sciences Ethics Committee (University of Exeter) and informed consent was obtained from all participants.

## Materials

To measure autistic-like traits, participants completed the 50-item adult Autistic Quotient (AQ: Baron-Cohen et al. 2001). The AQ assesses five sub-traits associated with ASD: attention to detail, attention switching, imagination, communication, and social skills. Participants self-reported on a four-point Likert scale, signalling whether they “definitely agree”, “slightly agree”, “slightly disagree”, or “definitely disagree” with 50 itemised statements assessing each subscale. Example statements include “I enjoy social occasions” (social skills), “I tend to notice details that others do not” (attentional switching) and “I am fascinated by dates” (attention to detail). The measure has proven reliable and valid for research use in general populations (Baron-Cohen et al. 2001; Woodbury-Smith et al. 2005), providing an overall score out of 50, whereby higher numbers reflect greater autistic tendencies. A score of 32 was proposed as a threshold above which seeking a diagnosis would be recommended for people who thought that they might be autistic (Baron-Cohen et al. 2001). As such, to reduce the possibility of relationships being driven by clinically related confounding factors (e.g., cognitive ability, symptom severity, development; Landry and Chouinard 2016), participants who recorded above this value were excluded from statistical analysis after they had completed the study (as in Buckingham et al. 2016).

**Fig. 1:**
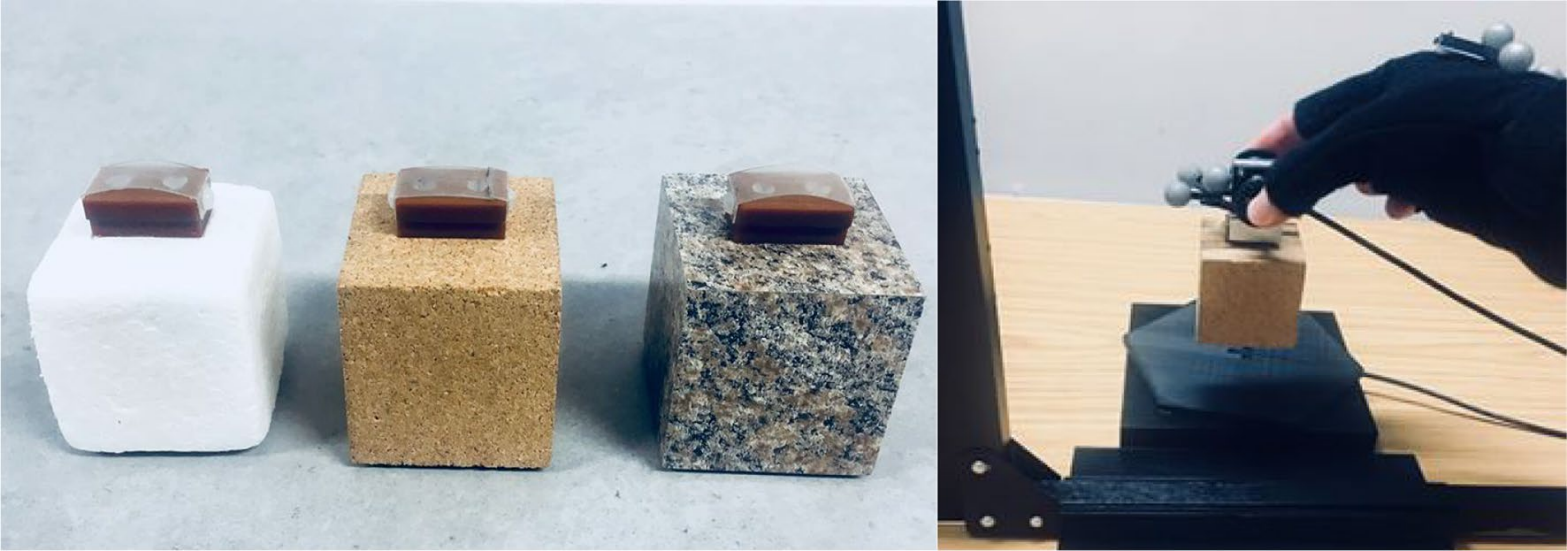
The expanded polystyrene, corkwood, and granite objects lifted by participants (**a**) and the experimental set-up during a lifting trial (**b**)

Participants were then presented with three identically sized (5 × 5 × 5 cm) cubes with different surface materials (Fig. 1a), namely: granite (unaltered density: 2.6 g/cm^3^), corkwood (unaltered density: 0.25 g/cm^3^), and expanded polystyrene (unaltered density: 0.03 g/cm^3^). Specifically, polystyrene (i.e., light-looking) and granite (i.e., heavy-looking) were used to elicit the MWI, whereas corkwood was selected to provide a ‘control’ object which was markedly closer to its natural (i.e., expected) weight. Each of the surface materials was sealed around a hollow wooden box, filled with lead shot and putty to provide a weight of 230 g. A clear adhesive was used to seal the surface material to its inner structure, thereby making the object appear completely made from its visible outer material. Care was taken to ensure that the centre of mass coincided with each object’s geometric centre.

A mount was positioned on each object’s top surface to facilitate lifting. Attached to this mount was an ATI Nano-17 Force transducer fitted within an aluminum and plastic handle (Fig. 1b), which recorded forces in three dimensions at 500 Hz. Grip force was defined by forces orthogonal to the handle’s surface, whereas load forces were yielded from the vector sum of the remaining values. Four reflective markers were attached to the object handle to create a detectable rigid body, which was tracked at 120 Hz by infrared cameras using motion-capture technology (OptiTrack Flex13, NaturalPoint, Corvallis, Oregon). Four markers were also positioned on a ‘lifting glove’ (Fig. 1b), which was worn on the dominant hand of participants to track hand movements.^1^ Participants were fitted with a Pupil Labs mobile eye-tracking system (Pupil Labs, Sanderstrasse, Berlin, Germany; Kassner et al. 2014), a pair of lightweight glasses (34 g) which collates information from scene and infrared eye cameras to calculate gaze positions at 90 Hz (spatial accuracy of ± 0.60° of visual angle; 0.08° precision). Prior to lifting procedures, the eye-tracking system was calibrated using the manufacturers built-in screen marker routine (Pupil Labs 2016), which was presented upon a large LED screen (60.96 cm; Dell Computer Corporation, Round Rock, TX, USA) that spanned the entire lifting workspace.^2^ Calibration procedures were repeated upon any displacement of gaze cameras. A chin rest was attached to the table to restrict head movements and a manual clapper board concealed objects before trials.

### Procedure

Participants first completed the AQ before undertaking the lifting protocol, consisting of 5 baseline lifts and 24 subsequent trials. Participants were seated throughout these trials, with their head positioned upon the chin rest, and were instructed to start with their dominant hand positioned to the side of the object. Each object was placed quietly in front of participants and concealed behind a closed clapper board until the onset of each trial, so that there was no prior indication of their properties. Upon a computer-generated auditory tone, the manual clapper board was opened to reveal an object, and participants reached out to grasp the lifting handle with their thumb and forefinger of their dominant hand. Participants were instructed to vertically lift the object in a ‘smooth, controlled, and confident manner’ at a self-selected speed, before holding it steady ‘a few centimetres above the table’. Upon a second auditory tone (+ 4 s after trial onset), they were required to gently place the object back in its starting position, before verbally reporting a numerical judgement about how heavy it felt. Apart from the condition, that larger numbers should represent higher weights, no constraints or ranges were placed on this measure so as to minimise biases associated with ratio scaling (Zwislocki and Goodman 1980).

Instructions of these standardised procedures were given ahead of the lifting protocol. Thereafter, the corkwood object was lifted five times, with participants informed that the object would not change during these baseline lifts. No procedural errors were displayed by participants during baseline lifts 3–5, suggesting that they were all familiar with the task requirements. Subsequent MWI trials consisted of lifting each object eight times, presenting a total of 24 lifts. The object used in each trial was determined from a completely randomised order, which was newly formulated for each participant to account for any potential order effects on weight perception (Maiello et al. 2018). Upon completion of all procedures, participants were verbally debriefed.

### Data analysis

#### Perceived heaviness scores

Self-ratings for each lift were normalised to a *z* score distribution to provide a measure of perceived heaviness. To quantify the magnitude of the experienced MWI, average values for the heavier-looking (granite) objects were subtracted from those of the lighter-looking (expanded polystyrene) objects (as in Buckingham et al. 2009, 2016).

#### Force data

Extracted data from the force transducers were processed and analyzed using a custom algorithm in MATLAB. Data were first smoothed using a 14-Hz dual-pass Butterworth filter, with forces perpendicular to the surface of the handle defined as grip force and resultant vectors of the tangential forces interpreted as load force (all as in Buckingham et al. 2009, 2016). To determine the rates of change, data were differentiated with a five-point central difference equation, with the maximum values on the initial lift for each trial determining peak grip (pGFR) and load (pLFR) force rates. Force rates from the first lift, as opposed to averages from all trials, were analyzed, as lifting forces adapt rapidly over repeated lifts (Flanagan and Beltzner 2000; Buckingham et al. 2009). To provide an index of prediction-led motor bias, grip (pGFRdiff) and load (pLFRdiff) force rates utilised in the first lift of the polystyrene object were subtracted from those of the granite object. Here, values from the first lift, as opposed to averages from across all lifts, were analyzed, as differences in lifting forces diminish rapidly over repeated lifts (Flanagan and Beltzner 2000; Buckingham et al. 2009).^3^ For these index scores, greater values would signify greater utility of feedforward information at a motor level.

#### Kinematic data

Positional data for each rigid body were smoothed using a dual-pass, zero-phase lag Butterworth filter at 10 Hz (the ‘optimum’ cut-off frequency reported for upper-limb movement control research; Franks et al. 1990). Hand and object velocities were calculated from the average position of their respected rigid bodies. We then segmented trials into four distinct phases: Reach, Grasp, Transport, and Hold (as in Lavoie et al. 2018). The reach phase started when hand velocity first exceeded 50 mm/s for three consecutive frames (Eastough and Edwards 2007) and concluded upon the onset of grip force (i.e., the Grasp phase). The Lift phase was then determined from the first timepoint whereby both Hand and Object velocity exceeded 50 mm/s. Finally, the Hold phase was derived from the timepoint where the object reached its maximum vertical position (endpoint of Lift phase) until the onset of the second auditory tone (trial completion). Total movement time was calculated from the onset of Reach to the offset of the Hold phase. The duration of each phase was recorded for baseline lifts and for the first lift of each MWI-inducing object. Furthermore, maximum velocity of the hand during reach (MRV) and lift (MLV) phases was recorded, as were the timepoints where this occurred (as a % of total movement time).

#### Gaze data

Visual fixations were extracted from gaze data using Pupil Player software (Pupil Labs 2016). Fixations were defined as a gaze that remained on a location (within 1° visual angle) for a minimum of 120 ms, with the total number and average duration of fixations recorded. To quantify visual search rate, the number of fixations was divided by the average fixation duration. To index ‘top–down’ control, we calculated the Quiet Eye (QE) duration, which represents the final fixation or tracking gaze before the initiation of a planned motor response (Vickers 1996). This was operationalised as the final fixation or tracking gaze directed to any single location in the workspace within 3° of visual angle (of the normalised position of the fixation’s centroid) for a minimum of 100 ms prior to the onset of the lift phase. These variables were assessed for baseline trials and for the first lift of each MWI object. Longer QE durations signify greater ‘top–down’ processing (Vine et al. 2014), whereas higher search rates are indicative of more stimulus-driven attention (Corbetta et al. 2008).

#### Eye–hand integration

To index the integration between gaze and kinematic outcomes, cross-correlational analysis (based on Chattington et al. 2007) explored the corresponding signals for the changes in the vertical component of eye and hand movement. First, positional hand data were resampled at 90 Hz, via interpolation, and gaze data were smoothed using a dual-pass, zero-phase lag Butterworth filter at 45-Hz (i.e., a low-pass cut-off deemed appropriate for detecting saccadic eye movements; Bahill et al. 1981). Thereafter, the two signals were manually synchronised for time, using detectable landmarks in the motion-capture and eye-tracking footage. Specifically, the frame denoting the onset of the reach movement was visually detected in the raw gaze data, before being aligned with the corresponding frame in the motion-capture data (i.e., where hand velocity first exceeds 50 mm/s for three consecutive frames). As the synchronised signals followed notably comparable profiles during the grasp, lift, and hold movements (see Online Appendix 1), data were then segmented from the start of the grasp phase (i.e., the timepoint corresponding to the onset of grip force) to the offset of the hold phase (i.e., the timepoint where the ‘object’ rigid body reached its maximum vertical position). The resulting cross-correlogram identified the peak covariation of the two signals (i.e., peak *R*) and the ‘lag’ (converted into time) for when this peak covariation occurred. This ‘lag’ measure quantified the degree to which one signal may lead another, with lower (i.e., more negative values), signifying that eye movements were preceding the hand to a greater extent in a more feedforward manner. This provided further insight into whether systems are integrated in a ‘top–down’ or ‘bottom–up’ manner (Chattington et al. 2007).

#### Preliminary analysis

Patterns of missing and complete values were identified for all data and the probability of these patterns diverging from randomness was estimated using Little’s MCAR test. To assist missing value analysis, Cronbach’s alpha coefficients assessed the reliability of AQ subscales. Outliers were inspected for all variables and, where detected, removed from their respected analysis (as recommended by Osbourne 2013). Here, univariate outliers were identified as values > 3.29 SD above or below the mean (*p* < 0.001) and multivariate outliers ascertained by extreme Mahalanobis distances (*p < *0.001; Tabachnick and Fidell 2007). Participants with > 10% of data identified as ‘missing’ or ‘outliers’ were excluded from analysis. For all variables, normality of data was examined from *z* scores for skewness and kurtosis, while assumptions relating to linearity, homoscedasticity, and multicollinearity were inspected from correlation matrices and scatterplots of residuals (Garson 2012).

#### Statistical analysis

To assess whether participants experienced the MWI and showed prediction-related motor patterns, separate three (polystyrene, corkwood, granite) × 8 (trials 1–8) repeated-measures ANOVAs were conducted, with pGFR, pLFR, and heaviness scores entered as dependent variables. Planned *t* tests using the Bonferroni correction probed significant effects, with effect sizes calculated using partial-eta squared ($\eta_{\text{p}}^{2}$). Pearson’s correlation examined relationships between AQ scores, perceptual MWI index scores and prediction-related measures of force (pGFRdiff, pLFRdiff), movement (grasp phase duration, MRV, MLV, and time to maximum velocity), gaze (search rate, QE duration), and eye–hand ‘lag’. Statistical analysis was performed using SPSS 25.0 for Windows (SPSS Inc., Chicago, IL, USA), with significance accepted for all the tests at *p* < 0.05 and data presented ± SD.

## Results

### Preliminary analyses

Incomplete cases were inferred as missing completely at random on the basis of Little’s MCAR test (*p* > 0.05), while Cronbach’s alpha coefficients indicated that AQ subscales were highly reliable (*α* > 0.70; Nunnally 1978). Consequently, missing AQ items (0.04%) were replaced using scale mean imputation and participants (*n* = 4) with > 10% of incomplete data were excluded from analysis. Three further participants were excluded due to “clinically significant” AQ scores (> 32) affording a final sample of 83. Remaining AQ scores ranged from 5 to 32 (Mean: 15.98 ± 6.60) and are, thus, comparable to Buckingham et al. (2016) previous data set (Mean: 15.41 ± 6.09). For sensorimotor outcomes, six participants were removed from force analysis (remaining *n *= 77), due to equipment malfunction and/or outlier analysis, while four participants were removed from kinematic analysis (remaining *n * = 79) and twenty from gaze analysis (remaining *n * = 63) due to poor data quality. There were no statistical violations relating to normality, homoscedasticity, and linearity observed on the remaining data. Mauchly’s test indicated that pGFR and pLFR violated the assumptions of sphericity (*p < *0.05) and the Greenhouse–Geisser correction was applied. No further modification or exclusion of variables was necessary. None of the perceptual or sensorimotor variables were significantly different between genders (all *p > *0.12) or left and right handers (*p*’s > 0.15; as in Buckingham et al. 2012).

### Primary analysis

#### Perceptual MWI

**Fig. 2:**
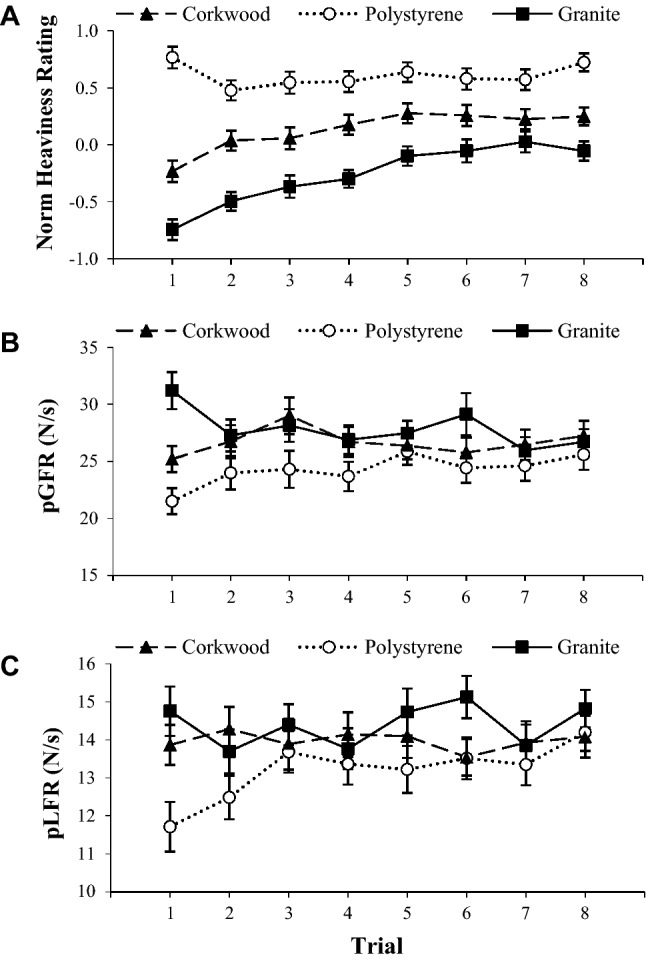
Trial-by-trial averages (± SEM) for normalised perceived heaviness ratings (**a**), peak grip force rate (pGRF; **b**), and peak load force rate (pLFR; **c**) across all trials

**Fig. 3:**
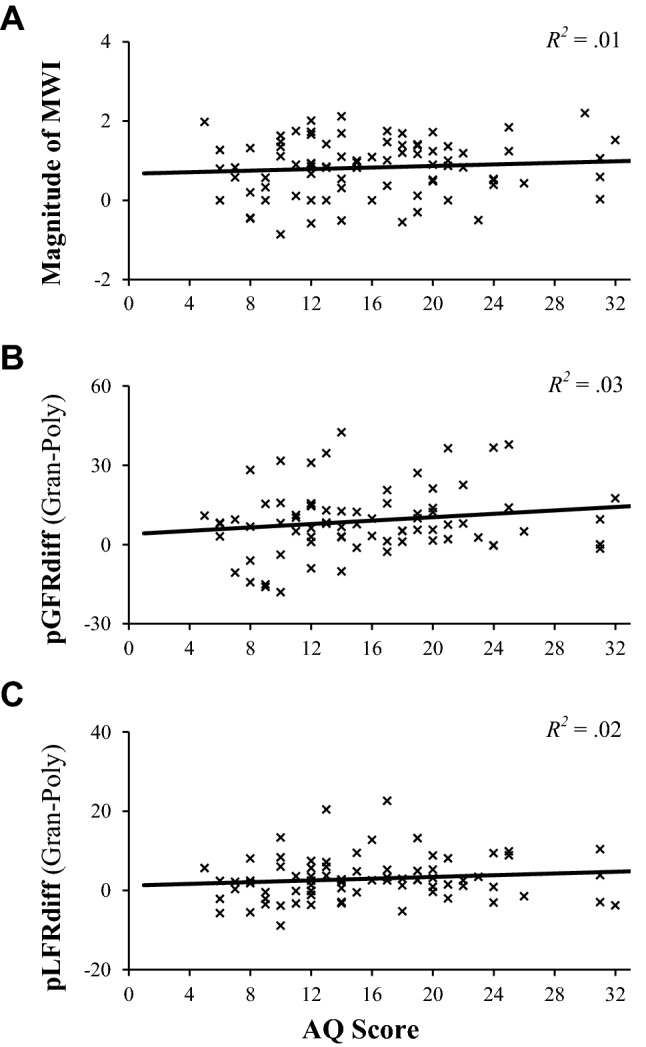
Scatter plots highlighting associations between AQ scores and the magnitude of the SWI (**a**), pGFRdiff (**b**), and pLFRdiff (**c**). No significant relationships emerged (all *p* > 0.05)

ANOVA revealed that a robust MWI was induced (Fig. 2A), with effects of material on perceived heaviness evident (*F*_(2, 162)_ = 59.57, *p* < 0.001, $\eta_{\text{p}}^{2}$ = 0.42). Average scores for the polystyrene object were greater than corkwood values (*t*_(82)_ = 5.42, *p* < 0.001), which, in turn, were significantly greater than those reported for the granite object (*t*_(82)_ = 5.38, *p* < 0.001). Surprisingly, a ‘material-by-trial’ interaction also emerged (*F*_(10.91, 883.43)_ = 3.54, *p* < 0.001, $\eta_{\text{p}}^{2}$ = 0.04), with the magnitude of the illusion greater on the initial lift of each object (Fig. 2a). Nevertheless, differences between materials were present during both initial (*F*_(2, 164)_ = 59.59, *p < *0.001, $\eta_{\text{p}}^{2}$ = 0.40) and final (*F*_(2,164)_ = 24.05, *p < *0.001, $\eta_{\text{p}}^{2}$ = 0.23) trials, suggesting that the MWI remained over the protocol. However, no significant associations between AQ scores and the magnitude of this effect emerged (*R* = 0.11; *p = *0.34; Fig. 3a), indicating that autistic-like traits are unrelated to one’s experience of this perceptual illusion.

#### Sensorimotor control

ANOVA revealed significant effects of object material on pGFR (*F*_(2,148)_ = 35.298, *p < *0.001, $\eta_{\text{p}}^{2}$ = 0.32) and pLFR (*F*_(2,144)_ = 18.09, *p* < 0.001, $\eta_{\text{p}}^{2}$* =* 0.20). As displayed in Fig. 2, fingertip forces were lower on the first trial when lifting the polystyrene box compared to when lifting the corkwood (pGFR: mean difference = 3.76 ± 8.05 N/s; *t*_(76)_ = 4.10, *p* < 0.001; pLFR: mean difference = 2.18 ± 4.01 N/s; *t*_(77)_ = 4.81, *p < *0.001) and granite (pGFR: mean difference = 10.04 ± 14.10 N/s; *t*_(77)_ = 6.29, *p* < 0.001; pLFR: mean difference = 3.31 ± 5.73 N/s; *t*_(76)_ = 5.06, *p* < 0.001) objects. Similarly, grip forces used to lift the granite box were significantly greater than those used to grip the corkwood one (pGFR: mean difference = 6.25 ± 12.33 N/s; *t*_(76)_ = 4.45, *p < *0.001), although pLFR were not significantly different between these objects (*t*_(76)_ = 1.05, *p* = 0.05). As expected, prediction-led biases in fingertip forces reduced over the lifting protocol (Fig. 2), suggesting that sensorimotor adaptation occurred. Therefore, these force data indicated that material-related weight expectancies biased motor control, particularly on the initial lifts of each object. However, pGFRdiff and pLFRdiff values were not significantly related to AQ scores (both *p > *0.15; Fig. 3), suggesting that this generic predictive bias of motor control is not linked to autistic tendencies. Furthermore, no significant relationships emerged between AQ scores and any gaze or kinematic indicators of predictive control (all *p > *0.12).

### Exploratory analysis

Naturally, effective predictive control of perception and action is dependent on accurate representations of environmental statistics (Friston 2005; Bastos et al. 2012), with ‘bottom–up’ attentional systems activated when uncertainty about one’s beliefs is high (Yu and Dayan 2003). However, recent theory (e.g., Lawson et al. 2014, 2017; Palmer et al. 2017) suggests that feedforward atypicalities in autism may arise from abnormalities in such processing. Therefore, given the null associations observed between autistic-like traits and broad indices of predictive control, we explored finer mechanisms relating to the context-sensitive integration of prior information and environmental statistics. Specifically, we indexed the degree to which AQ scores co-vary with uncertainty-related adjustments in gaze control, through subtracting average search rates in the final three baseline trials (i.e., where object properties were familiar and the likelihood of unexpected outcomes was minimal) from the first lift of each MWI object (i.e., where probabilistic and environmental statistics were uncertain).^4^

**Fig. 4:**
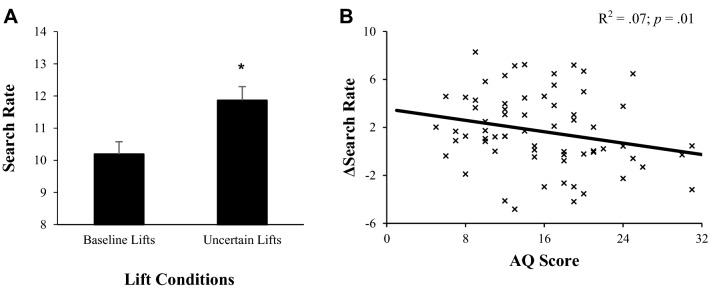
**a** Changes in search rate (± SEM) from baseline (lifts 3–5) to uncertain (initial MWI lifts for each object) lifts, and **b** scatter plot highlighting the relationship between AQ scores and the magnitude of these changes. *Denotes significant difference (*p* < 0.05)

As expected, search rate increased between baseline and MWI lifts (*t*_(62)_ = 4.24, *p* < 0.001), an effect driven by shorter fixation durations (average change: − 0.07 ± 0.13 s) which indicated that ‘bottom–up’ attentional systems were generally activated in uncertain trials (Fig. 4a). Interestingly though, these context-sensitive increases (i.e., differences in search rate between baseline and high-uncertainty trials) were negatively correlated with AQ scores (*R* = − 0.32, *p = *0.01), with more pronounced changes in low-trait compared to high-trait participants (Fig. 4b). This suggests that the utility of ‘top–down’ information was less flexible in those with greater autistic-like traits (Lawson et al. 2017).

Finally, given the possibility that some autistic-like traits may be more closely related to predictive processing than others, we explored relationships between individual AQ subscales and each of the sensorimotor outcomes included in the primary analysis (see Online Appendix 2 for Table). In line with our main findings, no associations emerged for any of force, kinematic, gaze, or perceptual variables (all *p* > 0.08), reinforcing observations that broad sensorimotor prediction is unrelated to autistic-like traits in the context of the MWI. Eye–hand ‘lag’ was observed to weakly correlate with ‘attention to detail’ (*R* = 0.26, *p = *0.045) and ‘attention switching’ (*R* = 0.28, *p = *0.03) subscales though, suggesting that there may be an association between visuomotor integration and autistic-like attentional traits.

## Discussion

In this study, we explored links between autistic-like traits and predictive sensorimotor control in a non-clinical population. To do this, we employed an MWI paradigm, whereby the apparent materials of identically sized and weighted objects were manipulated to elicit prediction-related patterns of perception, gaze, and movement. Manipulation checks indicated that prior expectations of object weight biased both perception and action (Fig. 2), permitting scrutiny into whether prediction-related tendencies are inherently related to autistic-like traits (e.g., as proposed by Pellicano and Burr 2012; Gomot and Wicker 2012; Friston et al. 2013; Sinha et al. 2014; Van de Cruys et al. 2014).

Contrary to our hypotheses, AQ scores were unrelated to the magnitude of the perceptual MWI (Fig. 3a), suggesting that the influence of prior knowledge on weight perception was comparable for individuals from across the general autism phenotype. These findings are difficult to reconcile with some predictive theories of autism (e.g., Pellicano and Burr 2012; Sinha et al. 2014) and illusory-based perceptual research (e.g., Happé 1996), as participants with greater AQ scores were equally susceptible to these prediction-led biases. However, they align with the null relationships observed between autistic-like traits and most classical illusions (Chouinard et al. 2016). In particular, our results indicate that the null perceptual effects observed by Buckingham et al. (2016) in the SWI were not specific to size processing mechanisms and hold true across a range of prior expectations.

Furthermore, and again contrary to our initial hypotheses, no broad-scale abnormalities in ‘top–down’ control of action were detected in high-trait individuals. Specifically, the extent to which prediction influenced motor patterns and gaze behaviors was unrelated to AQ scores, despite the previous findings that high-trait individuals utilise prior information differently in lifting motor programmes (Buckingham et al. 2016). Instead, participants generally displayed classic lifting profiles, irrespective of their AQ scores, whereby heavy-looking items were lifted with higher force rates than lighter-looking ones (Fig. 2b, c; Gordon et al. 1991; Flanagan and Beltzner 2000). Although seemingly contradictive of various sensorimotor research (e.g., Mosconi et al. 2013; Buckingham et al. 2016), this corresponds with a meaningful body of clinical evidence which has shown broad prediction-dependent capabilities to be typical in autistic people (Mostofsky et al. 2004; Gidley-Larson et al. 2008; Ego et al. 2016; Tewolde et al. 2018). Findings also align with recent evidence that autistic and neurotypical individuals attend to similar information when presented with visual illusions (Chouinard et al. 2018). Consequently, in contrast to broad predictive accounts of autism (e.g., Pellicano and Burr 2012; Sinha et al. 2014), our data indicate that links between sensorimotor prediction and autistic-like traits may not be due to any generic processing abnormalities, but rather due to context-sensitive ‘high-level’ mechanisms.

Recent theories propose that autistic-like traits may relate to finer mechanisms involved in the context-sensitive adjustment of ‘top–down’ and ‘bottom–up’ control systems (Lawson et al. 2014, 2017; Palmer et al. 2017). These contemporary accounts argue that prediction-related atypicalities may arise from implicit tendencies to misinterpret the uncertainty of an environment, with perception and action resting on internal representations of volatility (Friston 2005; Bastos et al. 2012). Typically, under more volatile conditions, less predictive attentional patterns emerge (Vossel et al. 2013), as evident in our data, where search rate generally increased between baseline and uncertain trials (Fig. 4a). This suppression of ‘top–down’ control is often adaptive, as prior expectations are less reliable in uncertain environments (Brown and Friston 2012), and resultant elevations in neural gain facilitate learning (Burge et al. 2008; Kording et al. 2007). Interestingly though, context-sensitive changes in search rate were reduced in high-trait participants (Fig. 4b), suggesting that the dynamic integration of prior information and environmental statistics may be decreased in these individuals. Although novel, such data are consistent with perceptual research, where high-trait participants showed reduced distinction between low- and high-volatility conditions (Lawson et al. 2017). They also correspond with the recent observations in the rubber-hand illusion, where participants with greater autistic-like traits displayed reduced uncertainty-related slowing of movement, despite experiencing typical perceptual effects (Palmer et al. 2013, 2015). Taken together, these results support proposals that predictive atypicalities in autism may stem from misrepresentations of environmental uncertainty (Lawson et al. 2014, 2017).

These contemporary explanations account for why feedforward differences are shown in some, but not all empirical paradigms, as environmental statistics will naturally vary. For example, it is plausible that context-sensitive representations of uncertainty differed in the present MWI study from Buckingham et al. (2016) SWI protocol, where the congruity between expected and actual weight will have differed. Furthermore, given the “finer”, “context-sensitive” predictive processes implicated by these theoretical frameworks (Palmer et al. 2017, p 521), quantifiable differences in sensorimotor control are unlikely to transfer across SWI and MWI lifting paradigms, as they are underpinned by different mechanisms (Buckingham 2014; Saccone and Chouinard 2019). Nevertheless, various autism-related movement difficulties can be explained by heightened perceptions of volatility, with motor skill performance (Land 2009) and adaptation (Burge et al. 2008) both impaired by contextually inappropriate weightings of ‘top–down’ and ‘bottom–up’ control. Therefore, given the growing evidence for these explanations, research should explore the effects of environmental volatility on sensorimotor control in autism. The use of weight-based illusions to further this understanding remains profitable, as they facilitate holistic exploration of sensorimotor control in a manner that is not contingent upon communicative or motivational competencies (Fisk and Goodale 1989).

Currently, our findings must be interpreted with caution in the context of clinical populations (Gregory and Plaisted-Grant 2016), with inferences essentially indirect at this stage (Skewes et al. 2015). Although trait-based approaches are advocated in the recent research (Chouinard et al. 2013), motor impairments are more prevalent and/or severe in clinical populations (Green et al. 2002) and may, thus, differ in aetiology. Further research is consequently required to examine whether results hold in individuals with clinically diagnosed ASD, to assist in the development of evidence-based practical interventions that are warranted by autistic stakeholders and representative organisations (Pellicano et al. 2014; Myers and Johnson 2007). Indeed, it is argued that greater scrutiny into prediction-related mechanisms, such as those discussed in here, could present numerous avenues for prospective diagnostic and treatment programmes (see Haker et al. 2016 for detailed discussion). Though it must be emphasised that our study provides only a tentative starting point in this research development, it is hoped that future work will be directed towards helping autistic people “manage themselves with whatever difficulties they have” (Pellicano et al. 2014, p 6).

It must also be noted that the simplistic nature of our motor task may limit the validity of ‘eye–hand’ measures. As the goal of each trial was to assess object weight, deviations in ‘top–down’ and ‘bottom–up’ mechanisms were difficult to detect, with the objects providing an informational source for both attentional systems. Thus, unsurprisingly, ‘eye–hand’ lag times (0.23 ± 0.09) were temporally closer than those previously observed (e.g., Lavoie et al. 2018), as gaze tended to follow the object in a manner that aids perception of weight (Hamilton et al. 2007). Interestingly, sub-trait analysis (Online Appendix 2) suggested that this integration of visuomotor systems may be related to autistic-like attentional traits. This exploratory link evidently requires further empirical scrutiny, with heightened perceptions of volatility proposed to disrupt the ‘connectivity’ of neurobiological systems (Friston et al. 2013). More sophisticated eye–hand analysis is warranted during tasks with an external goal (e.g., Lavoie et al. 2018), where eye movements typically precede those of the hand in an empirically quantifiable fashion (Chattington et al. 2007). Such enquiry could improve our understanding of how sensory domains might be related in autism (Robertson and Baron-Cohen 2017).

Overall, our findings suggest that sensorimotor atypicalities in people with greater autistic tendencies do not originate from domain-general processing impairments, but rather from specific differences in the utility of predictive control. Participants with greater autistic-like traits appeared equally susceptible to predictive biases elicited by the MWI at multiple sensorimotor levels. However, these individuals showed reduced context-sensitive adjustments in gaze control under uncertain conditions, supporting links between autistic-like traits and inflexible representations of environmental volatility. Research is required to further our mechanistic understanding of these effects and enable the development of effective evidence-based strategies for the autism community.

## Electronic supplementary material

Below is the link to the electronic supplementary material.

Supplementary material 1 (DOCX 27 kb)

## Footnotes

1 Although there were initial concerns over whether these reflective markers would artificially disrupt participants’ gaze during trials, raw data indicated that participants rarely, if ever fixated on these features. Such observations are reinforced by a recent object interaction study (Lavoie et al. 2018), where identical motion-capture and gaze registration systems showed that participants rarely fixate on ‘marked’ anatomical regions (e.g., the hand) and instead focus on task-relevant cues (e.g., the objects and prospective lifting paths). Therefore, we are confident that this issue did not confound our gaze data.

2 Pilot gaze positional data showed minimal variance in the *z-*axis (i.e., depth), with attention almost entirely deployed towards the current and future object position (as in Johansson et al. 2001). As participants were only instructed to move the object in the vertical plane, no corrections were deemed necessary to account for the altered geometry of the three-dimensional workspace. Instead, the calibration screen monitor was placed exactly at the location of the ‘lifting platform’, so that gaze could be specifically calibrated in relation to the expected visual workspace.

3 This rapid trial-by-trial adaptation is not shown in relation to the perceptual MWI, where erroneous perceptions of heaviness remain unchanged throughout extended protocols (Buckingham et al. 2009).

4 Search rate, as opposed to QE duration, was selected as our index of gaze control, as there was greater between-subject variance (i.e., individual differences) in this measure at baseline. Furthermore, the index measure was hypothesised to encapsulate the context-sensitive activation of *‘bottom–up’* attentional systems that emerge in uncertain environments (Yu and Dayan 2003; Vossel et al. 2013).

## References

[CR1] Almeida RA, Dickinson JE, Maybery MT, Badcock JC, Badcock DR (2014). Enhanced global integration of closed contours in individuals with high levels of autistic-like traits. Vis Res.

[CR2] Bahill AT, Brockenbrough A, Troost BT (1981). Variability and development of a normative data base for saccadic eye movements. Invest Ophthalmol Vis Sci.

[CR3] Baron-Cohen S, Wheelwright S, Skinner R, Martin J, Clubley E (2001). The autism-spectrum quotient (AQ): evidence from asperger syndrome/high-functioning autism, males and females, scientists and mathematicians. J Autism Dev Disord.

[CR4] Baron-Cohen S, Hoekstra RA, Knickmeyer R, Wheelwright S (2006). The Autism-Spectrum Quotient (AQ)—adolescent version. J Autism Dev Disord.

[CR5] Bastos AM, Usrey WM, Adams RA, Mangun GR, Fries P, Friston KJ (2012). Canonical microcircuits for predictive coding. Neuron.

[CR9] Brandwein AB, Foxe JJ, Butler JS, Frey H-P, Bates JC, Shulman LH, Molholm S (2015). Neurophysiological indices of atypical auditory processing and multisensory integration are associated with symptom severity in autism. J Autism Dev Disord.

[CR11] Brosnan MJ, Scott FJ, Fox S, Pye J (2004). Gestalt processing in autism: failure to process perceptual relationships and the implications for contextual understanding. J Child Psychol Psychiatry.

[CR03] Brown H, Friston KJ (2012). Free-energy and illusions: the cornsweet effect. Front Psychol.

[CR13] Buckingham G (2014). Getting a grip on heaviness perception: a review of weight illusions and their probable causes. Exp Brain Res.

[CR14] Buckingham G, Goodale MA (2013). Size matters: a single representation underlies our perceptions of heaviness in the size-weight illusion. PLoS One.

[CR15] Buckingham G, Cant JS, Goodale MA (2009). Living in a material world: how visual cues to material properties affect the way that we lift objects and perceive their weight. J Neurophysiol.

[CR16] Buckingham G, Ranger NS, Goodale MA (2012). Handedness, laterality and the size-weight illusion. Cortex.

[CR17] Buckingham G, Michelakakis EE, Rajendran G (2016). The influence of prior knowledge on perception and action: relationships to autistic traits. J Autism Dev Disord.

[CR18] Buescher AV, Cidav Z, Knapp M, Mandell DS (2014). Costs of autism spectrum disorders in the United Kingdom and the United States. JAMA Pediatr.

[CR19] Burge J, Ernst MO, Banks MS (2008). The statistical determinants of adaptation rate in human reaching. J Vis.

[CR22] Charpentier A (1891). Analyse experimentale de quelques elements de la sensation de poids. Archive de Physiologie Normale et Pathologiques.

[CR23] Chattington M, Wilson M, Ashford D, Marple-Horvat D (2007). Eye–steering coordination in natural driving. Exp Brain Res.

[CR25] Chouinard PA, Noulty WA, Sperandio I, Landry O (2013). Global processing during the Müller-Lyer illusion is distinctively affected by the degree of autistic traits in the typical population. Exp Brain Res.

[CR26] Chouinard PA, Unwin KL, Landry O, Sperandio I (2016). Susceptibility to optical illusions varies as a function of the autism-spectrum quotient but not in ways predicted by local–global biases. J Autism Dev Disord.

[CR013] Chouinard PA, Royals KA, Landry O, Sperandio I (2018). The Shepard illusion is reduced in children with an autism spectrum disorder because of perceptual rather than attentional mechanisms. Fronti Psychol.

[CR28] Cooper NR, Simpson A, Till A, Simmons K, Puzzo I (2013). Beta event-related desynchronization as an index of individual differences in processing human facial expression: further investigations of autistic traits in typically developing adults. Front Hum Neurosci.

[CR29] Corbetta M, Patel G, Shulman GL (2008). The reorienting system of the human brain: from environment to theory of mind. Neuron.

[CR32] Eastough D, Edwards MG (2007). Movement kinematics in prehension are affected by grasping objects of different mass. Exp Brain Res.

[CR33] Ego C, Bonhomme L, de Xivry J-JO, Da Fonseca D, Lefèvre P, Masson GS, Deruelle C (2016). Behavioral characterization of prediction and internal models in adolescents with autistic spectrum disorders. Neuropsychologia.

[CR015] Fisk JD, Goodale MA (1989). The effects of instructions to subjects on the programming of visually directed reaching movements. J Mot Behav.

[CR35] Flanagan JR, Beltzner MA (2000). Independence of perceptual and sensorimotor predictions in the size–weight illusion. Nat Neurosci.

[CR38] Fournier KA, Hass CJ, Naik SK, Lodha N, Cauraugh JH (2010). Motor coordination in autism spectrum disorders: a synthesis and meta-analysis. J Autism Dev Disord.

[CR39] Franks IM, Sanderson DJ, Van Donkelaar P (1990). A comparison of directly recorded and derived acceleration data in movement control research. Hum Mov Sci.

[CR012] Friston K (2005). A theory of cortical responses. Philos Trans Royal Soc B Biol Sci.

[CR40] Friston KJ, Lawson R, Frith CD (2013). On hyperpriors and hypopriors: comment on Pellicano and Burr. Trends in Cognitive Sciences.

[CR41] Frith C (2003). What do imaging studies tell us about the neural basis of autism. Autism: neural basis and treatment possibilities: Novartis foundation symposium.

[CR05] Fuentes CT, Mostofsky SH, Bastian AJ (2009). Children with autism show specific handwriting impairments. Neurology.

[CR42] Garson GD (2012). Testing statistical assumptions.

[CR43] Geisler WS, Kersten D (2002). Illusions, perception and Bayes. Nat Neurosci.

[CR45] Gernsbacher MA, Stevenson JL, Dern S (2017). Specificity, contexts, and reference groups matter when assessing autistic traits. PLoS One.

[CR46] Gidley-Larson JC, Bastian AJ, Donchin O, Shadmehr R, Mostofsky SH (2008). Acquisition of internal models of motor tasks in children with autism. Brain.

[CR49] Gomot M, Wicker B (2012). A challenging, unpredictable world for people with autism spectrum disorder. Int J Psychophysiol.

[CR50] Gordon AM, Forssberg H, Johansson RS, Westling G (1991). Visual size cues in the programming of manipulative forces during precision grip. Exp Brain Res.

[CR51] Gowen E, Hamilton AF (2013). Motor abilities in autism: a review using a computational context. J Autism Dev Disord.

[CR52] Grandy MS, Westwood DA (2006). Opposite perceptual and sensorimotor responses to a size-weight illusion. J Neurophysiol.

[CR53] Green D, Baird G, Barnett AL, Henderson L, Huber J, Henderson SE (2002). The severity and nature of motor impairment in Asperger’s syndrome: a comparison with specific developmental disorder of motor function. J Child Psychol Psychiatry.

[CR54] Gregory B, Plaisted-Grant K (2016). The autism-spectrum quotient and visual search: shallow and deep autistic endophenotypes. J Autism Dev Disord.

[CR56] Haker H, Schneebeli M, Stephan KE (2016). Can Bayesian theories of autism spectrum disorder help improve clinical practice?. Front Psychiatry.

[CR57] Hamilton AFDC, Flanagan DW, Frith JR, Wolpert DM (2007). Kinematic cues in perceptual weight judgement and their origins in box lifting. Psychol Res.

[CR58] Happé FG (1996). Studying weak central coherence at low levels: children with autism do not succumb to visual illusions. A research note. J Child Psychol Psychiatry.

[CR61] Haswell CC, Izawa J, Dowell LR, Mostofsky SH, Shadmehr R (2009). Representation of internal models of action in the autistic brain. Nat Neurosci.

[CR63] Jameel L, Vyas K, Bellesi G, Roberts V, Channon S (2014). Going ‘above and beyond’: are those high in autistic traits less pro-social?. J Autism Dev Disord.

[CR64] Jasmin E, Couture M, McKinley P, Reid G, Fombonne E, Gisel E (2009). Sensori-motor and daily living skills of preschool children with autism spectrum disorders. J Autism Dev Disord.

[CR67] Johansson RS, Westling G (1988). Coordinated isometric muscle commands adequately and erroneously programmed for the weight during lifting task with precision grip. Exp Brain Res.

[CR68] Johansson RS, Westling G, Bäckström A, Flanagan JR (2001). Eye–hand coordination in object manipulation. J Neurosci.

[CR69] Johnson BP, Rinehart NJ, White O, Millist L, Fielding J (2013). Saccade adaptation in autism and Asperger’s disorder. Neuroscience.

[CR70] Kassner M, Patera W, Bulling A (2014) Pupil: an open source platform for pervasive eye tracking and mobile gaze-based interaction. In: Adjunct Proceedings of the 2014 ACM international joint conference on pervasive and ubiquitous Computing (UbiComp), pp 1151–1160

[CR02] Kording KP, Tenenbaum JB, Shadmehr R (2007). The dynamics of memory as a consequence of optimal adaptation to a changing body. Nat Neurosci.

[CR71] Land MF (2009). Vision, eye movements, and natural behavior. Vis Neurosci.

[CR04] Landry O, Chouinard PA (2016). Why we should study the broader autism phenotype in typically developing populations. J Cogn Dev.

[CR72] Lavoie EB, Valevicius AM, Boser QA, Kovic O, Vette AH, Pilarski PM, Chapman CS (2018). Using synchronized eye and motion tracking to determine high-precision eye-movement patterns during object-interaction tasks. J Vis.

[CR73] Lawson RP, Rees G, Friston KJ (2014). An aberrant precision account of autism. Front Human Neurosci.

[CR74] Lawson RP, Mathys C, Rees G (2017). Adults with autism overestimate the volatility of the sensory environment. Nat Neurosci.

[CR01] Leary MR, Hill DA (1996). Moving on: autism and movement disturbance. Ment Retard.

[CR75] Maiello G, Paulun VC, Klein LK, Fleming RW (2018). The sequential-weight illusion. i-Perception.

[CR76] Mitchell P, Mottron L, Soulieres I, Ropar D (2010). Susceptibility to the Shepard illusion in participants with autism: reduced top-down influences within perception?. Autism Res.

[CR77] Mosconi MW, Luna B, Kay-Stacey M, Nowinski CV, Rubin LH, Scudder C, Sweeney JA (2013). Saccade adaptation abnormalities implicate dysfunction of cerebellar-dependent learning mechanisms in autism spectrum disorders (ASD). PLoS One.

[CR79] Mostofsky SH, Bunoski R, Morton SM, Goldberg MC, Bastian AJ (2004). Children with autism adapt normally during a catching task requiring the cerebellum. Neurocase.

[CR80] Myers SM, Johnson CP (2007). Management of children with autism spectrum disorders. Pediatrics.

[CR011] Nunnally J (1978). Psychometric theory.

[CR010] Osbourne JW (2013). Best practices in data cleaning: a complete guide to everything you need to do before and after collecting your data.

[CR81] Palmer CJ, Paton B, Hohwy J, Enticott PG (2013). Movement under uncertainty: the effects of the rubber-hand illusion vary along the nonclinical autism spectrum. Neuropsychologia.

[CR82] Palmer CJ, Paton B, Kirkovski M, Enticott PG, Hohwy J (2015). Context sensitivity in action decreases along the autism spectrum: a predictive processing perspective. Proc R Soc B Biol Sci.

[CR83] Palmer CJ, Lawson RP, Hohwy J (2017). Bayesian approaches to autism: towards volatility, action, and behavior. Psychol Bull.

[CR84] Pellicano E, Burr D (2012). When the world becomes ‘too real’: a Bayesian explanation of autistic perception. Trends Cogniti Sci.

[CR85] Pellicano E, Dinsmore A, Charman T (2014). What should autism research focus upon? Community views and priorities from the United Kingdom. Autism.

[CR86] Poljac E, Poljac E, Wagemans J (2013). Reduced accuracy and sensitivity in the perception of emotional facial expressions in individuals with high autism spectrum traits. Autism.

[CR87] Pupil Labs (2016) *Pupil Labs.* Retrieved from www.pupil-labs.com/. Accessed 01 Nov 2018

[CR88] Robertson CE, Baron-Cohen S (2017). Sensory perception in autism. Nat Rev Neurosci.

[CR89] Ropar D, Mitchell P (2002). Shape constancy in autism: the role of prior knowledge and perspective cues. J Child Psychol Psychiatry.

[CR90] Ruzich E, Allison C, Smith P, Watson P, Auyeung B, Ring H, Baron-Cohen S (2015). Measuring autistic traits in the general population: a systematic review of the Autism-Spectrum Quotient (AQ) in a nonclinical population sample of 6,900 typical adult males and females. Mol Autism.

[CR06] Saccone EJ, Chouinard PA (2019). The influence of size in weight illusions is unique relative to other object features. Psychon Bull Rev.

[CR92] Scharoun SM, Wright KT, Robertson-Wilson JE, Fletcher PC, Bryden PJ (2017) Physical activity in individuals with autism spectrum disorders (ASD): a review. In: Autism-paradigms, recent research and clinical applications: InTech. https://www.intechopen.com/books/autism-paradigms-recent-research-and-clinical-applications/physical-activity-inindividuals-with-autism-spectrum-disorders-asd-a-review

[CR93] Schmitz C, Martineau J, Barthélémy C, Assaiante C (2003). Motor control and children with autism: deficit of anticipatory function?. Neurosci Lett.

[CR94] Seashore CE (1899). Some psychological statistics II. The material weight illusion. Univ Iowa Stud Psychol.

[CR95] Sinha P, Kjelgaard MM, Gandhi TK, Tsourides K, Cardinaux AL, Pantazis D, Held RM (2014). Autism as a disorder of prediction. Proc Natl Acad Sci.

[CR016] Skewes JC, Jegindø EM, Gebauer L (2015). Perceptual inference and autistic traits. Autism.

[CR96] Soulières I, Dawson M, Samson F, Barbeau EB, Sahyoun CP, Strangman GE, Mottron L (2009). Enhanced visual processing contributes to matrix reasoning in autism. Hum Brain Mapp.

[CR97] Tabachnick BG, Fidell LS (2007). Using multivariate statistics.

[CR98] Tewolde FG, Bishop DV, Manning C (2018). Visual motion prediction and verbal false memory performance in autistic children. Autism Res.

[CR101] Van de Cruys S, Evers K, Van der Hallen R, Van Eylen L, Boets B, de-Wit L, Wagemans J (2014). Precise minds in uncertain worlds: predictive coding in autism. Psychol Rev.

[CR102] Van der Hallen R, Evers K, Brewaeys K, Van den Noortgate W, Wagemans J (2015). Global processing takes time: a meta-analysis on local–global visual processing in ASD. Psychol Bull.

[CR103] Vickers JN (1996). Visual control when aiming at a far target. J Exp Psychol Hum Percept Perform.

[CR09] Vine SJ, Moore LJ, Wilson MR (2014). Quiet eye training: the acquisition, refinement and resilient performance of targeting skills. Eur J Sport Sci.

[CR014] Vossel S, Mathys C, Daunizeau J, Bauer M, Driver J, Friston KJ, Stephan KE (2013). Spatial attention, precision, and Bayesian inference: a study of saccadic response speed. Cereb Cortex.

[CR105] Wang Z, Magnon GC, White SP, Greene RK, Vaillancourt DE, Mosconi MW (2015). Individuals with autism spectrum disorder show abnormalities during initial and subsequent phases of precision gripping. J Neurophysiol.

[CR08] Williams AM, Singer RN, Frehlich SG (2002). Quiet eye duration, expertise, and task complexity in near and far aiming tasks. J Mot Behav.

[CR07] Wolfe HK (1898). Some effects of size on judgments of weight. Psychol Rev.

[CR107] Woodbury-Smith MR, Robinson J, Wheelwright S, Baron-Cohen S (2005). Screening adults for Asperger syndrome using the AQ: a preliminary study of its diagnostic validity in clinical practice. J Autism Dev Disord.

[CR108] Yu A, Dayan P (2003). Uncertainty, neuromodulation, and attention. Neuron.

[CR109] Zwislocki J, Goodman D (1980). Absolute scaling of sensory magnitudes: a validation. Percept Psychophys.

