# Supplementary material for: Exploring how material cues drive sensorimotor prediction across different levels of autistic-like traits
Source: Exp Brain Res. 2019 Jun 27;237(9):2255–67. doi: 10.1007/s00221-019-05586-z (PMC6675774; doi:10.1007/s00221-019-05586-z)
Supplement: Supplementary file 1 — Supplementary material 1 (DOCX 27 kb) [file 221_2019_5586_MOESM1_ESM.docx]

**Appendix 1: Time-synchronised signals of the vertical component of eye and hand movements** **during a baseline lifting trial.** Data taken from a single participant’s (ID: 38) first baseline lift. Time adjusted relative to grasp phase onset.

**
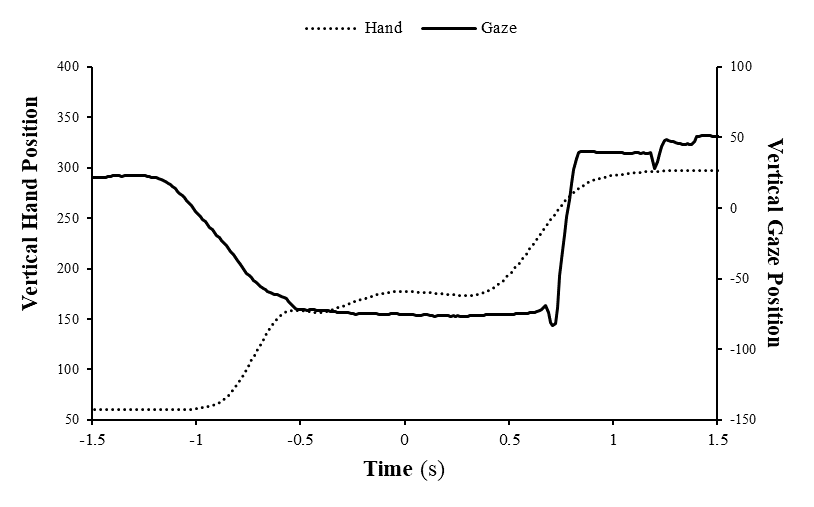
**

*Note*: Following grasp phase onset (time = 0), positional signals follow comparable vertical profiles, with hand movements slightly ‘leading’ changes in gaze position (Peak *R* = 0.60; ‘Lag’ = 0.11).

**Appendix 2: Exploratory Analysis.**

| **Supplementary Table 1.** Bivariate correlations between sensorimotor outcomes and sub-traits assessed in the 50-item Autistic Quotient. | | | | | | |
| --- | --- | --- | --- | --- | --- | --- |
|  | | **AQ Subscales** | | | | |
|  | Social Skills | | Attention Switching | Attention to Detail | Communication | Imagination |
| MWI Magnitude | 0.21 | | 0.13 | -0.06 | 0.14 | 0.04 |
| pGFRdiff | 0.06 | | 0.17 | 0.09 | 0.08 | 0.13 |
| pLFRdiff | 0.01 | | 0.06 | 0.07 | 0.16 | 0.15 |
| Grasp Time | -0.11 | | -0.11 | -0.16 | -0.20 | -0.01 |
| MRV | 0.05 | | 0.04 | -0.10 | -0.06 | -0.05 |
| MLV | 0.19 | | 0.01 | 0.16 | 0.18 | -0.02 |
| Time to MRV | -0.04 | | 0.06 | 0.04 | 0.10 | -0.12 |
| Time to MLV | -0.14 | | -0.14 | 0.04 | -0.06 | 0.03 |
| Search Rate | 0.07 | | 0.15 | -0.13 | 0.11 | -0.09 |
| QE duration | -0.01 | | -0.08 | 0.14 | -0.15 | 0.02 |
| Eye-Hand ‘lag’ | 0.04 | | 0.26^*^ | 0.28^*^ | 0.05 | 0.03 |
| AQ: Autistic Quotient; MWI: Material-Weight Illusion; pGFRdiff: difference in grip force rate; pLFRdiff: difference in load force rate; MRV: maximum reach velocity; MLV: maximum lift velocity; QE: quiet eye;  *^*^ p* < .05 | | | | | | |
